# Quantitative Identification of Mutant Alleles Derived from Lung Cancer in Plasma Cell-Free DNA via Anomaly Detection Using Deep Sequencing Data

**DOI:** 10.1371/journal.pone.0081468

**Published:** 2013-11-21

**Authors:** Yoji Kukita, Junji Uchida, Shigeyuki Oba, Kazumi Nishino, Toru Kumagai, Kazuya Taniguchi, Takako Okuyama, Fumio Imamura, Kikuya Kato

**Affiliations:** 1 Research Institute, Osaka Medical Center for Cancer and Cardiovascular Diseases, Osaka, Osaka, Japan; 2 Department of Thoracic Oncology, Osaka Medical Center for Cancer and Cardiovascular Diseases, Osaka, Osaka, Japan; 3 Graduate School of Informatics, Kyoto University, Kyoto, Japan; 4 PRESTO, JST, Uji, Kyoto, Japan; Memorial Sloan Kettering Cancer Center, United States of America

## Abstract

The detection of rare mutants using next generation sequencing has considerable potential for diagnostic applications. Detecting circulating tumor DNA is the foremost application of this approach. The major obstacle to its use is the high read error rate of next-generation sequencers. Rather than increasing the accuracy of final sequences, we detected rare mutations using a semiconductor sequencer and a set of anomaly detection criteria based on a statistical model of the read error rate at each error position. Statistical models were deduced from sequence data from normal samples. We detected epidermal growth factor receptor (*EGFR*) mutations in the plasma DNA of lung cancer patients. Single-pass deep sequencing (>100,000 reads) was able to detect one activating mutant allele in 10,000 normal alleles. We confirmed the method using 22 prospective and 155 retrospective samples, mostly consisting of DNA purified from plasma. A temporal analysis suggested potential applications for disease management and for therapeutic decision making to select epidermal growth factor receptor tyrosine kinase inhibitors (EGFR-TKI).

## Introduction

For some molecular targeted drugs against cancer, the examination of genomic changes in target genes has become a diagnostic routine and is indispensable for treatment decisions. For example, the strong effects of epidermal growth factor receptor tyrosine kinase inhibitors (EGFR-TKIs; i.e., gefitinib and erlotinib) on non-small-cell lung cancer (NSCLC) are correlated with activating somatic mutations in *EGFR* [1,2]. Patients who are administered these drugs are currently selected based on the presence of these activating mutations. The identification of the mutations is based on biopsy samples; the procedure is invasive and often difficult to perform. A non-invasive diagnostic procedure is desirable. 

 Cell-free DNA in the blood consists of DNA derived from cancer tissues and has been studied for non-invasive diagnostic procedures [3]. This DNA, termed circulating tumor DNA (ctDNA), is rare in blood, and its detection is a technical challenge. A number of methods have been examined, but most of them have limitations in sensitivity and robustness. BEAMing (beads, emulsion, amplification and magnetics) [4] is most likely the most sensitive method. In BEAMing, PCR products amplified from a single molecule are fixed to a single magnetic bead using emulsion PCR. The mutation site is labeled with a fluorescent probe or primer extension, and the mutated allele is quantitatively detected by counting the fluorescently labeled beads. BEAMing successfully quantified *APC* and *KRAS* mutations in the ctDNA of colorectal cancer patients [5,6] and *EGFR* mutations in the ctDNA of lung cancer patients [7]. In spite of its high sensitivity and quantification ability, BEAMing has not gained in popularity because it is a laborious technology and requires oligonucleotides for each mutation position. 

Because BEAMing and next-generation sequencers, i.e., massively parallel sequencers, use the same or a very similar template preparation technique, it is possible to apply next-generation sequencers for the same purpose. There have been several studies on the deep sequencing of cell-free DNA [8,9]. These studies suggested the possibility of the approach but lacked critical evaluation of the detection systems. In particular, they did not address the problem of multiple testing, which is inherent to diagnostic applications. 

 In this report, we established a method of detecting *EGFR* mutations in ctDNA in the peripheral blood of lung cancer patients using single-pass deep sequencing of amplified *EGFR* fragments. The recent development of a semiconductor sequencer (Ion Torrent PGM) [10] has addressed the shortcomings of other currently available sequencers (i.e., a long runtime for a single assay and high operating costs) and is applicable for diagnostic purposes. We applied anomaly detection [11,12] and determined a set of detection criteria based on a statistical model of the read error rate at each error position. The method quantitatively detected *EGFR* mutations in cell-free DNA at a level comparable to BEAMing, promising non-invasive diagnostics that complement biopsy.

## Results

### Principle of detection

Deep sequencing of a PCR-amplified fragment containing a mutation site can be conducted to detect and quantitate mutated alleles among the vast amounts of normal alleles derived from host tissues. The major problem associated with this approach is the frequency of errors introduced during sequencing and PCR amplification. The key issue here is the setting and accurate evaluation of detection limits. When the frequency of a base change at a target locus is higher than a predetermined read error rate (RER), we may judge the change to be due to the presence of a mutant sequence. That is, anomalies that fall significantly outside of the RER distribution are regarded as mutations. The RER is defined as the error rate calculated from final sequence data, including errors in both the sequencing and PCR steps. In anomaly detection [11,12], as in hypothesis testing, false positives are controlled based on a statistical model. In our case, the statistical model of the RER can be constructed from sequence data from the target regions of a sufficient number of normal individuals carrying no mutations. 

**Figure 1:**
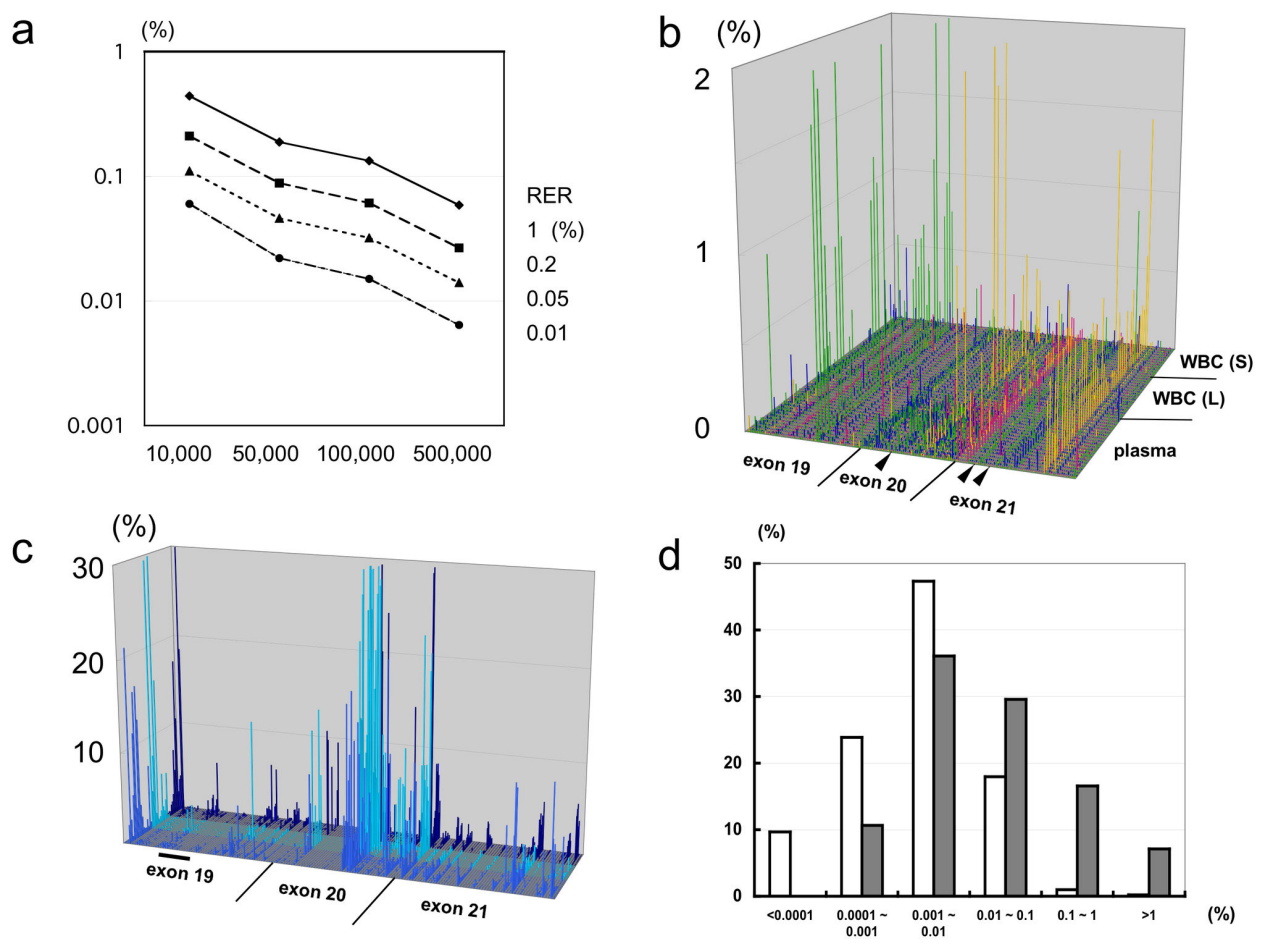
Read error of Ion Torrent PGM in the *EGFR* target region. a, Relationship between the read error rate, read depth, and detection limit for mutations when the significance level is p=2x10^-5^. Horizontal axis, read depth; vertical axis, detection limit (%). From top to bottom, each line indicates a read error rate (RER) of 1%, 0.2%, 0.05%, or 0.01%. b, Three-dimensional representation of substitution RER. x-axis, base positions of EGFR exons 19–21. From left to right, the arrowheads indicate the positions of T790M, L858R, and L861Q. y-axis, 48 DNA samples from normal individuals. From front to back, conversions to A (green), C (yellow), G (magenta), or T (blue) are aligned for each sample. z-axis, RER (%). c, Three-dimensional representation of the insertion/deletion error. x-axis, base positions of EGFR exons 19–21. The bar indicates the position of the exon 19 deletion. y-axis, 48 DNA samples from normal individuals. Blue, plasma DNA; light blue, WBC DNA (large amount); dark blue, WBC DNA (small amount). z-axis, RER (%). d, Distribution of the RER. White column, substitution error; gray column, insertion/deletion error. Horizontal axis, range of RER (%); vertical axis, incidence (%).

 If read errors occur under a probability distribution, the number of reads required to achieve a certain detection limit can be estimated. Figure 1a shows the relationship between the mutation detection limit, read depth, and RER at a significance level of p=2x10^-5^ for each individual detection without multiplicity correction, assuming that read errors occur following a Poisson distribution. The data illustrated in Figure 1a are supplied in Table S1. With an increasing read depth and decreasing RER, the detection limit decreases. In a previous study by our group [7], the detection limit for rare mutant alleles when using BEAMing [4] was 1 in 10,000 (0.01%). Because a plasma DNA assay sample contains approximately 5,000 molecules, this detection limit is reasonable. This goal can be achieved with 100,000 reads when the RER is below 0.01%. 

### Read error of the *EGFR* target region

For EGFR-TKI treatment, an activating *EGFR* mutation is indicative of treatment efficacy [1,2]. Patients to be administered these drugs are currently selected based on the presence of these activating mutations. In addition to activating *EGFR* mutations, a resistant *EGFR* mutation known as T790M appears in approximately half of patients subjected to EGFR-TKI treatment [13,14]. Thus, three activating mutations, i.e., a deletion in *EGFR* exon 19 and L858R and L861Q in *EGFR* exon 21, as well as the T790M resistant mutation in *EGFR* exon 20 were selected as target loci. 

We determined the RERs in a 169 base region around the target loci consisting by performing deep sequencing of DNA samples from normal individuals. We used an Ion Torrent PGM [10] sequencer for this work. Single-pass sequencing was performed, and the number of reads ranged from 44,400 to 373,000, averaging 162,000. We employed three types of DNA samples: 19 plasma DNA samples with amounts comparable to patients’ samples, 16 leucocyte (white blood cell, WBC) DNA samples with amounts that were 10 or 50 times the size of a patient’s sample, and 13 WBC DNA samples with amounts that were one-tenth the size of a patient’s sample. We divided substitution errors into four patterns, corresponding to conversion to A, C, G, or T. Thus, there were 507 possible types of substitutions (169 base positions x 3 patterns) in the target region. A substitution RER is graphically shown in Figure 1b, excluding the conversion from G to A at position 2,361 due to a frequent SNP. The substitution RERs are not uniform, nor are they independent from each other, and high RERs are associated with specific base positions. In addition, one substitution pattern is dominant at each base position. An insertion/deletion RER is graphically shown in Figure 1c. We did not distinguish between deletion and insertion errors, as insertions are often recognized as deletions and vice versa by the sequence alignment software. The insertion/deletion RER is generally higher than the substitution RER. A tendency similar to that of substitution is observed, in that high insertion/deletion RERs are associated with specific base positions. Figure 1d presents the distribution of the RERs. There were substantial differences between the substitution and insertion/deletion RERs. In 410 out of the possible 506 types of substitution (81.0%), the RER was lower than 0.01%. In contrast, out of the 169 types of insertions/deletion, the RER was lower than 0.01% in only 79 (46.7%). These results agreed with previously reported observations from the PGM platform [15]. The data illustrated in Figures 1b and 1c are supplied in Tables S2 and S3, respectively. 

Due to high insertion/deletion read errors, we employed a specific method to detect the exon 19 deletion mutations. We prepared eight template exon 19 sequences with representative deletions and screened the deletion sequences by matching them with the template sequences. This method was quite effective for screening out read errors; no sequences with deletion read errors were found among the 48 samples tested. 

### Statistical models of read error rates and criteria for anomaly detection

**Figure 2:**
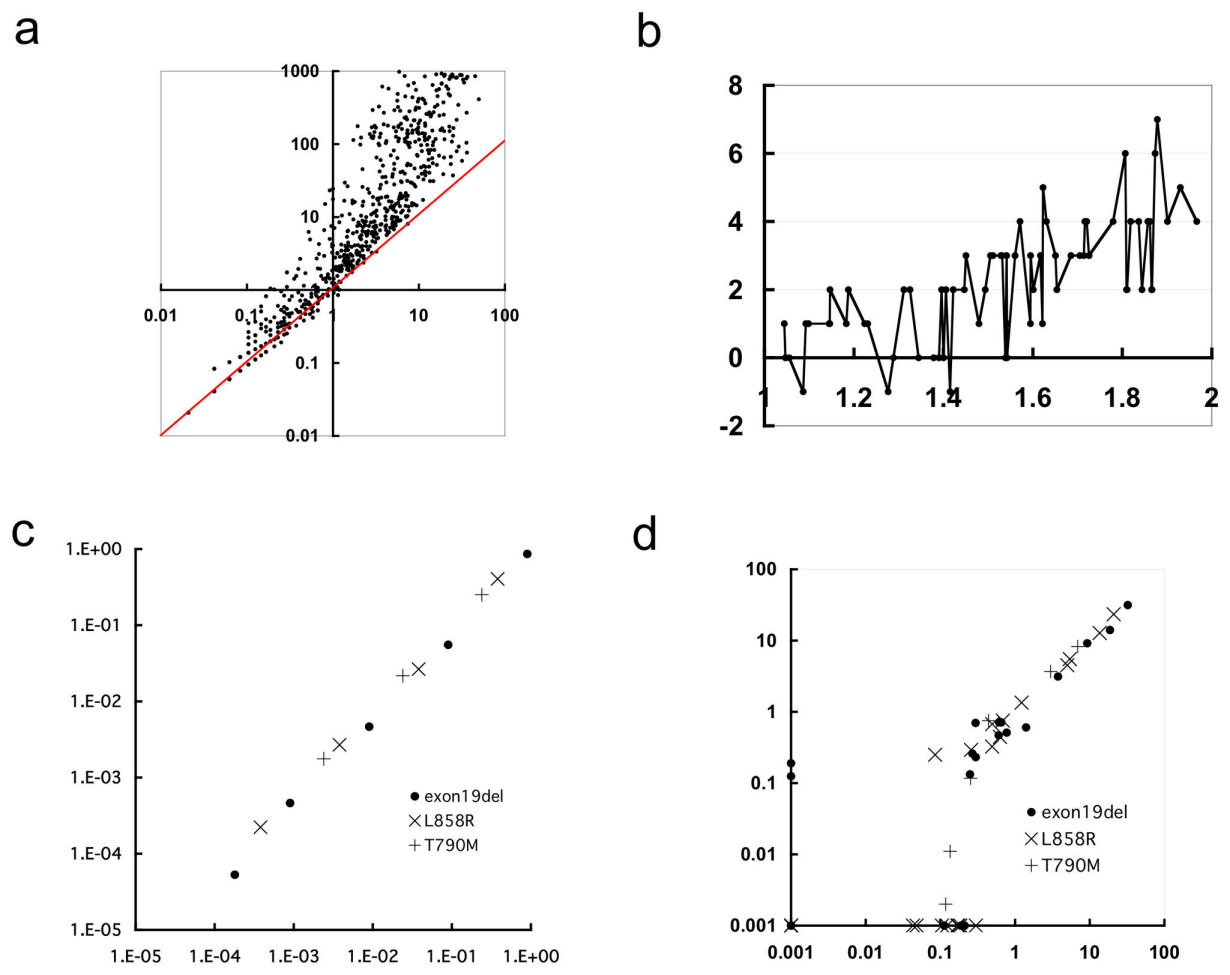
Characteristics of the mutation detection system. a, Relationship between the average and variance of the substitution error presented as the number per 100,000 reads. Horizontal axis, average; vertical axis, variance. The red line indicates where the average and variance are equal. b, Difference between thresholds calculated according to a negative binomial distribution and a Poisson distribution. The threshold is the minimum number of base changes in 100,000 reads meeting the level of statistical significance (p-0.01). Horizontal axis, variance/average ratio of the substitution read error; vertical axis, difference between thresholds. The types of substitutions whose variance/average ratio ranged from 1 to 2 are plotted. c, Accuracy of quantitation. Each data point represents the average of three assays. Horizontal axis, fraction of mutant alleles in artificial products; vertical axis, fraction of mutant alleles estimated from deep sequencing. d, Reproducibility of quantitation. Horizontal axis, base change rate in the first trial; vertical axis, base change rate in the second trial.

We then examined statistical models of read error. In a Poisson distribution model, the average and variance of the number of incidences are expected to be the same and are determined by the intensity parameter *lambda*. Here, instead of using the RER, the read error incidence was presented as the incidence in 100,000 reads, and its average and variance at each base position were calculated. The relationships between the average and variance are shown in Figure 2a and Figure S1 in File S1 for the substitution and insertion/deletion read errors, respectively. In both cases, the variance becomes greater than the average in a considerable proportion of the cases. In these cases, application of the Poisson distribution would lead to increased numbers of false positives. This phenomenon, termed “overdispersion”, is common in biological studies, and in such cases, a negative binomial distribution is applied [16]. Overdispersion is due to fluctuations of the intensity parameter, and it is rational to assume that the intensity parameter follows a gamma distribution. Under this scenario, the incidence number theoretically follows a negative binomial distribution. In Figure 2b, the increase in the threshold for substitution from a Poisson to a negative binomial distribution is plotted against the variance/average ratio of the read error for the substitution types whose variance/average ratio ranged from 1 to 2. When the ratio exceeded approximately 1.2-1.4, there were substantial increases in threshold. Thus, we constructed our statistical model of each substitution under the following criteria. 

1 When the average read error in 100,000 reads was less than 1, a Poisson distribution with λ set to 1 was applied (169 types of substitutions).
2 When the average was greater than 1 and the variance/average ratio of the read error was less than 1.2, a Poisson distribution was applied (15 types of substitutions).
3 When the average was greater than 1 and the variance/average ratio of the read error was greater than 1.2, a negative binomial distribution was applied (323 types of substitutions).

The exon 19 deletion and L858R belonged to the first category, while the L861Q and T790M mutation sites belonged to the second and the third categories, respectively. The detection limits for the exon 19 deletion and the L858R, L861Q, and T790M substitution mutations at a significance level of p=2x10^-5^ were less than 0.01% and less than 0.01%, 0.01%, and 0.05%, respectively. In the following analysis, we used p=2x10^-5^ as the significance threshold for each single detection, without considering a multiplicity correction, expecting one false positive in 50,000 samples.

 The outline of the method is 1) amplification of *EGFR* fragments with exon-specific primers from plasma DNA; 2) deep sequencing of *EGFR* fragments with PGM (>100,000 reads / fragment), combining the PCR products; 3) matching the output sequences with *EGFR* template sequences; 4) detection of deletions and substitutions, and conversion of number of events into that in 100,000 reads; and 5) evaluation of the base changes with the anomaly detection criteria. In anomaly detection, the base changes are judged as mutations, when the number of events in 100,000 reads is equal to or exceeds the threshold value (exon 19 deletion, 7; L858R, 7; L861Q, 12; T790M, 60). A schematic representation is shown in Figure S2 in File S1.

### Quantitativity and reproducibility

First, we examined the method’s quantification ability. We prepared test samples including various fractions of PCR products of mutated *EGFR* fragments. There was a very good linearity (*r*=0.998) between the inoculated amounts of the PCR products and the observed mutant-to-normal allele ratios deduced from deep sequencing (Figure 2c). We then examined the reproducibility of the method using plasma samples from lung cancer patients whose primary lesions were confirmed to carry activating mutations. The fractions of the mutant alleles measured in two trials are plotted in Figure 2d. A high concordance (*r*=0.989) was observed, except in samples that contained small amounts of the mutant alleles, corresponding to an approximately 0.3% fraction of the alleles present or less. In these cases, the initial phase of PCR amplification was likely to be unsuccessful due to the low numbers of mutant templates, estimated at 15 copies or less. Thus, the limit of quantitation was approximately 0.3%. 

### Validation with samples from lung cancer patients

We further evaluated our method using lung cancer biopsy specimens, sampling plasma DNA and the primary lesion simultaneously as part of a prospective study. The results for the samples from 22 patients showed 86% concordance (95% confidence interval, 66 - 95), 78% (44 - 93) sensitivity, and 92% (66 - 98) specificity, setting the tissue biopsy as the standard. These results are promising with respect to the development of a diagnostic tool to complement lung cancer biopsy. 

We then analyzed a total of 155 samples: 144 samples from plasma, eight from cerebrospinal fluid, and one each from urine, pleural effusion, and bronchial alveolar lavage. As for plasma samples, two or more samples were obtained from 32 patients at different time points of the disease courses. All of the obtained data are shown in Table S4. Clinical data of the patients including stage, histology, treatment, and status of resistance to EGFR-TKI are also listed in this Table. Among the 33 patients associated with a primary lesion containing the exon 19 deletion, this mutation was found in at least one of the plasma samples from 24 patients (72.7%). Of the 23 patients for which the primary lesions exhibited the L858R or L861Q substitutions, these mutations were found in at least one of the plasma samples from 18 patients (78.2%). A double mutation (simultaneous detection of the exon 19 deletion and L858R) was observed in 12 plasma samples, although double mutations are not frequent in biopsy samples. Discrepancies between the activation mutation types identified in biopsy and plasma DNA samples were observed in five plasma samples. T790M was found in 13 out of 57 plasma samples (22.8%) from patients with EGFR-TKI resistance, and in 7 out of 87 plasma samples (8.0%) without EGFR-TKI resistance. 

### Temporal changes of *EGFR* mutation levels during the disease course

**Figure 3:**
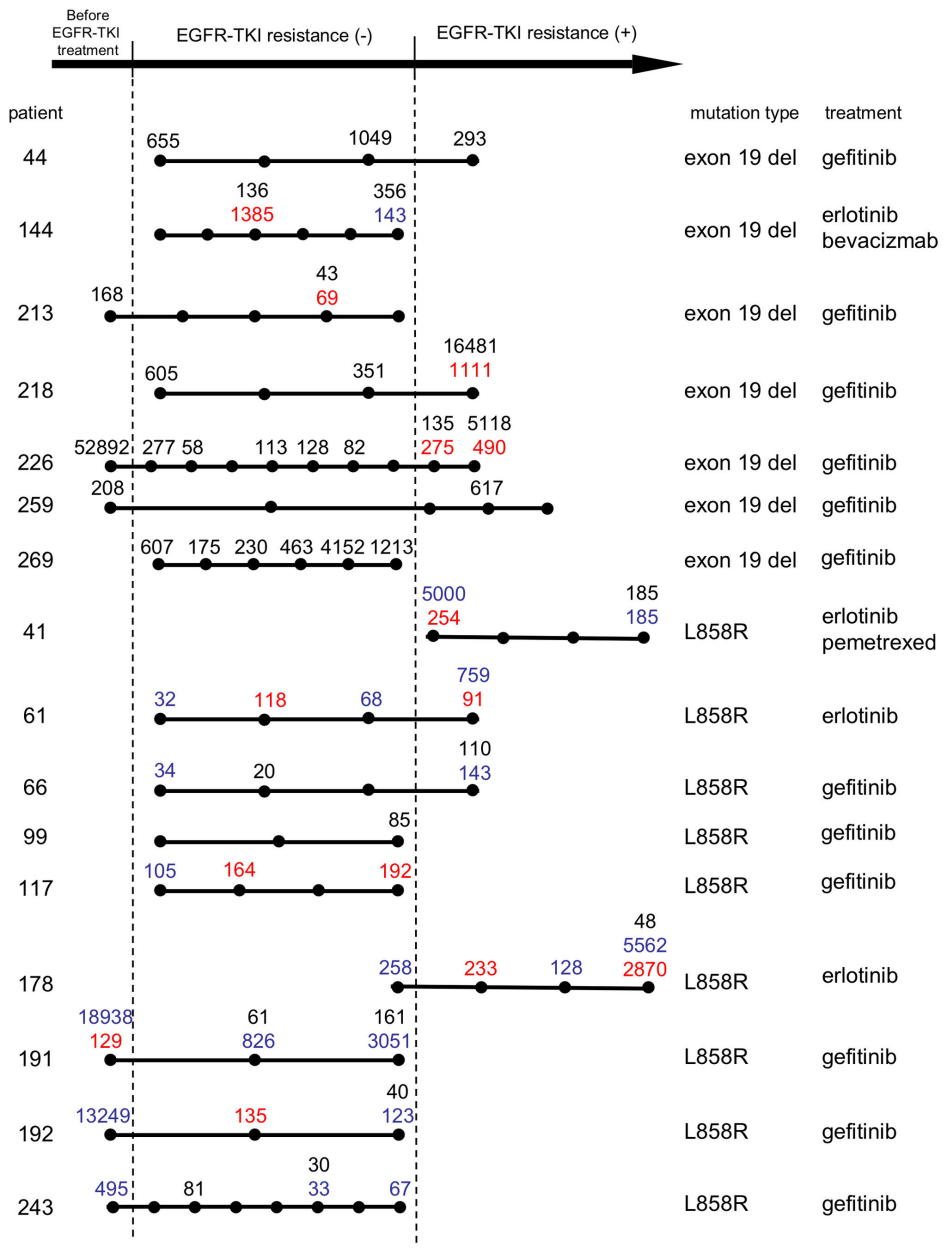
Temporal changes of *EGFR* mutations in plasma DNA from patients with three or more samples. Each dot represents a time point of sampling. The diagram is not precise representation of time scale, and only the order of dots is valid information. Figures represent *EGFR* mutations in 10,000 sequence reads: black, exon 19 deletion; blue, L858R; red, T790M. Only figures exceeding the thresholds are shown. “Mutation type” indicates that in the biopsy samples.

A considerable number of samples were collected from the same patient at different time points in the disease course. Temporal changes of *EGFR* mutation levels in plasma DNA from patients with three or more samples are schematically shown in Figure 3. Due to the relatively short sampling period, samples were obtained from only part of the disease course in most cases. We focused on two transitions: transition due to EGFR-TKI treatment initiation and that after acquiring EGFR-TKI resistance. Data before the treatment initiation was obtained in six cases. A significant decrease in the activation of mutation levels with the treatment was seen in all cases (p=1.7x10^-4^). Clearance of ctDNA by the treatment initiation is a general phenomenon. 

 Data were obtained both before and after acquiring EGFR-TKI resistance in seven cases. After acquiring resistance, the activation of mutation level was increased in five patients (218, 226, 259, 61, 66), decreased in one patient (44), and increased with delay in another patient (178). Increase of activation of mutations may correlate with disease progression. Despite the clear correlation between T790M and the EGFR-TKI-resistance status in the above validation study, dynamics of T790M during the disease course was not as clear as that of activation of mutations; T790M often appeared before acquiring resistance.

Three patients are described in more detail. Patient 226 was treated with gefitinib as first line chemotherapy. The gefitinib treatment was stopped several times due to adverse effects. A radiological response (partial response, PR) was observed from month 1 to month 9, and disease progression was observed in month 10. Prior to gefitinib treatment, the fraction of the mutant allele was very high (>50%), but after only one week of this treatment, the fraction of the mutant allele decreased to 0.3%, prior to any radiological changes (Figure S3a in File S1). T790M appeared at 10 months when disease progression began. Patient 243 also exhibited a skewed decrease in the mutant allele fraction at the initiation of gefitinib treatment (Figure S3b in File S1). This patient was treated with surgery and adjuvant chemotherapy (CDDP plus VNR) previously, and then subjected to gefitinib. Patient 41 presented with progression of neoplastic meningitis, and was subjected to combined erlotinib-pemetrexed therapy. Previous treatments were CDDP plus gemicitabine, gefitinib, and erlotinib. A minor radiological response was observed from months one to four, and disease progression occurred subsequently. There was a skewed decrease in the mutant allele fraction at the beginning of the therapy, and the increase upon disease progression was only slight (Figure S3c in File S1). It should be noted that the respose of ctDNA to EGFR-TKI treatment initiation was rapid in all three cases (patient 229, one week; 243, two weeks; 41, one month). 

### Mutation detection in the entire target region

**Figure 4:**
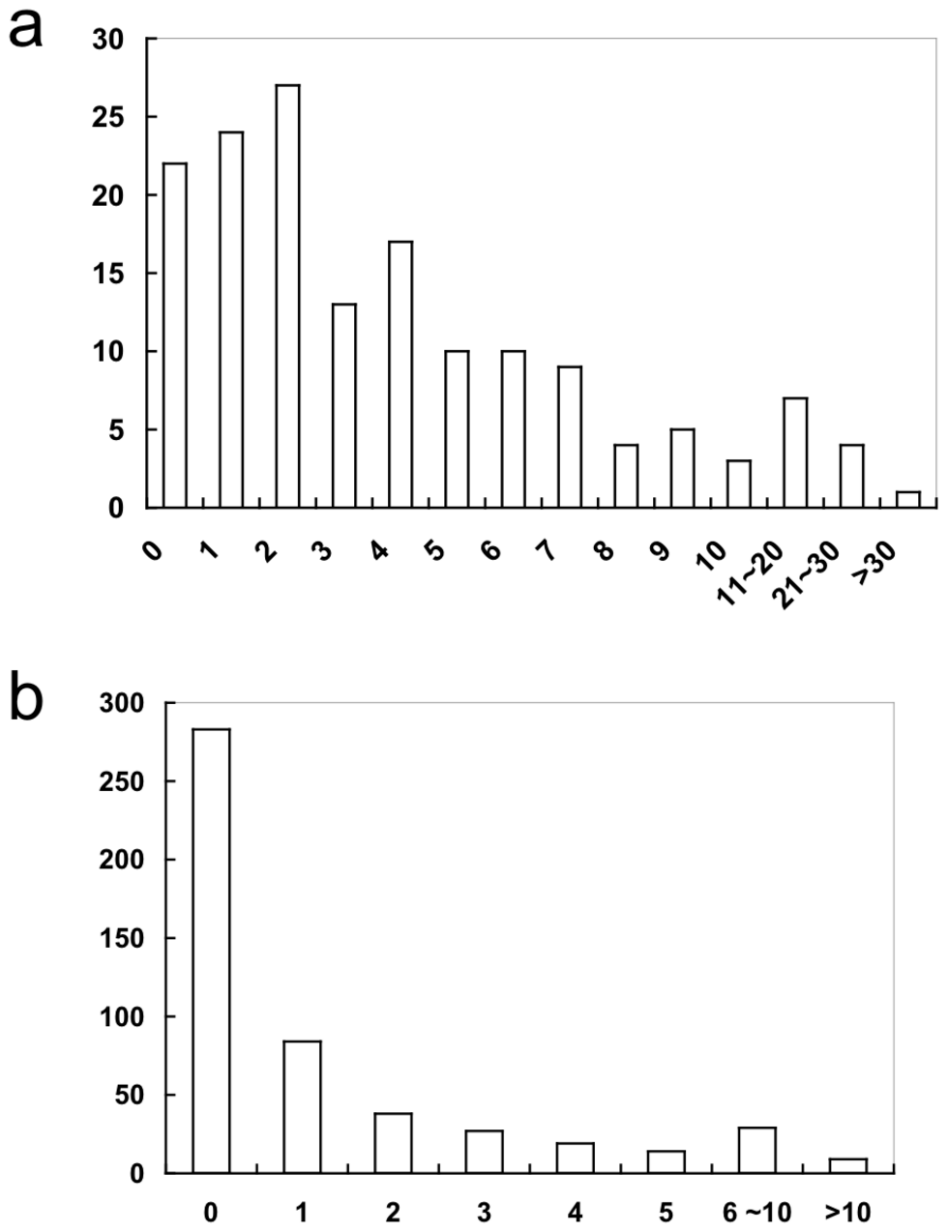
Substitutions introduced in output sequences of the 155 retrospective samples. a, Distribution of the number of different types of substitutions judged as mutations per sample. Horizontal axis, number of the types of substitutions; vertical axis, number of samples. b, Distribution of the number of samples with a substitution type judged as a mutation. Horizontal axis, the number of samples with a substitution type judged as a mutation; vertical axis, number of the types of substitutions.

We explored the possibility of identifying substitution mutations in the entire target *EGFR* region corresponding to 503 types of substitutions, excluding L858R, L861Q, and T790M. Because the significance level was set at p=2x10^-5^ for each detection, false positives were expected to appear once in 100 samples. In reality, a median of three substitutions were found per sample. The distribution of the number of substitutions per sample is shown in Figure 4a. Based on the experience gained from the biopsy samples, most of these substitutions were likely to be false positives. A considerable fraction of the different types of substitutions presented no false positives (56.2%, Figure 4b), and the statistical models were of practical use with these types of substitutions. For others, the parameter estimation from the data from 48 normal individuals was not sufficiently conservative for the exclusion of false positives. 

## Discussion

Rare mutation detection of target loci through the deep sequencing of plasma cell-free DNA has a comparable sensitivity to BEAMing. The specificity is also acceptable because the *EGFR* mutation types in biopsy and plasma samples exhibited a high concordance. Thus, rare mutation detection with deep sequencing has now reached a sufficient level to proceed to confirmation through a prospective study. The method could be applied to a limited number of target loci at any base position; using the pair-end method or sequencing from the opposite direction would increase the accuracy of high error rate positions, increasing sensitivity and specificity to acceptable levels. 

 However, it is difficult to extend mutation detection to a larger region. The incidence of false positives is not acceptable for diagnostic applications. Parameter estimation with increased numbers of normal samples and/or more conservative estimation methods, such as Bayesian inference, might decrease false positives. We used mutation-free DNA from normal individuals for the survey of read error, but mutation detection was performed with plasma DNA from lung cancer patients. A possible cause of the inadequate thresholds may be the difference in DNA quality. The recent discovery of artifactual mutations introduced during experimental processes [17] suggests the possibility of still undiscovered causes of artifacts using plasma samples. 

Our procedure is optimized for our objectives and social environment, but there is room for technical improvement. In addition to the paired-end method [9], methods to produce error-free sequences through the repeated sequencing of templates from a single molecule [18,19] might be applicable to enhance accuracy. We employed small amounts of plasma DNA for PCR amplification due to the ethical standards of our hospital and relevant regional hospitals. However, in a different social environment, using an increased amount of plasma DNA may improve the reproducibility of the detection of low-level mutations. 

 In addition to being applied for the non-invasive diagnosis of *EGFR* mutations, as shown in the above temporal analyses, this method is also informative for elucidating the dynamics of mutant alleles during the course of the disease. In particular, it should be noted that a skewed decrease in the mutant allele fraction preceded radiological changes, which will likely be useful for the prediction of drug efficacy. 

 Biopsies of advanced cases and repeated biopsies are technically demanding, and replacement with a non-invasive method would be beneficial. In this context, monitoring T790M with our method would have substantial benefits for patient management. For example, detecting the T790M mutation in blood samples would be useful for patient selection for treatment with new EGFR-TKIs for lung cancers that are resistant to gefitinib and erlotinib [20].

 Recent two studies suggest other possibilities of ctDNA analysis. Dawson et al. followed the dynamics of ctDNA in metastatic breast cancer patients using mutations in *TP53* and/or *PIK3CA*, and found its merit for monitoring disease progression [21]. Use of common mutations may enable its application to a wide variety of tumors. On the contrary, our research focus is more specific, i.e., mutation detection for therapeutic decision making, although our method can also be applied for their purpose. Murtaza et al. performed exome sequencing using plasma DNA from cancer patients [22], Analysis of cancer genomes at any stage of the disease course might uncover genetic changes leading to disease progression or drug resistance. Analysis of ctDNA will have a profound value in scientific and diagnostic aspects of cancer research.

## Materials and Methods

### Patient characteristics

Patients with activating EGFR mutations in tumor tissues were recruited at Osaka Medical Center for Cancer and Cardiovascular Diseases. Pleural fluid, cerebrospinal fluid and/or urine samples were collected from some patients. In all of the patients, activating *EGFR* mutations were found in biopsy samples using the PNA-LNA PCR clamp method [23]. The response to therapy and disease progression were mainly evaluated from radiological data based on the RECIST criteria [24]. 

### DNA extraction from liquid samples

Plasma was prepared via centrifugation of 4-5 ml of EDTA-treated blood at 800 *g* for 10 min at room temperature. The plasma was transferred to a fresh tube and re-centrifuged at 15,100 *g* for 10 min at room temperature. After centrifugation, the upper plasma was transferred to a fresh tube. Pleural fluid and urine samples were centrifuged at 800 *g* for 10 min at room temperature, and the supernatants were transferred to fresh tubes. Centrifuged liquid samples were frozen at -80 °C until DNA extraction. Cerebrospinal fluid was frozen without centrifugation. DNA was extracted from 1.5–2.0 ml of a liquid sample (or 5 ml of urine) using the QIAamp circulating nucleic acid kit (Qiagen, Hilden, Germany) according to the manufacturer’s instructions. The DNA concentration was determined by measuring the copy number of LINE-1 [25] or using the Qubit ssDNA Assay Kit (Life Technologies, Carlsbad, CA, USA). 

### Amplicon library construction and deep sequencing

#### Sequencing library construction

To amplify target regions of the *EGFR* gene, PCR primer pairs were designed with Primer3 (http://frodo.wi.mit.edu/). Primer pairs have 5-nt indexes (to discriminate individuals) and adaptor sequences for semiconductor-sequencing. Positions of PCR-target regions and primer sequences are shown in Table S5. PCR amplification was conducted in a 50 µl reaction mixture containing plasma DNA obtained from 300 µl of plasma (10 ng or more), 20 pmol of each primers and 1 unit of KOD -Plus- DNA polymerase (Toyobo, Osaka, Japan). To analyze the read error, we used genomic DNA from plasma or leukocytes from healthy individuals as a PCR template. The cycling profile was as follows: 2 min at 94°C for initial denaturation, followed by 40 cycles of 15 sec at 94°C for denaturation, 30 sec at 55°C for annealing, and 50 sec at 68 °C for extension. The products were purified using the QIAquick 96 PCR Purification Kit (Qiagen) or the MinElute PCR Purification Kit (Qiagen), and the DNA concentration was determined using the Quant-iT™ PicoGreen® dsDNA Assay Kit (Life Technologies) or an ND-1000 Spectrophotometer (NanoDrop Technologies, Montchanin, DE, USA). Subsequently, we mixed equal volumes of the purified PCR products and diluted them to create a template for emulsion PCR. We mixed 12 and 24 types of PCR products for use with Ion 316 and Ion 318 semiconductor chips, respectively. 

#### Semiconductor sequencing

Sequencing template preparation (emulsion PCR and beads-enrichment) from sequencing libraries was carried out using an Ion OneTouch Template Kit (Life Technologies) and Ion OneTouch system (Ion OneTouch Instrument and Ion OneTouch ES, Life Technologies) according to manufacturer’s protocol. Prepared templates were sequenced using Ion Sequencing Kit v2 and the Personal Genome Machine (Life Technologies). Number of nucleotide flows during sequencing was set to 200 (50 cycles). Torrent Suite 2.2 (Life Technologies) was used for converting raw signals into base calls, and extracting FASTQ files of sequencing reads. Read depth for one assay mostly exceeded 100,000. Sequencing data were deposited in DDBJ Sequence Read Archive (accession number: DRA001029).

### Counting variants

#### Alignment of sequencing reads

Reads in FASTQ files were divided using 5-nt indexes for individual assignment using in-house perl script. Short reads (<70 bases) were discarded. Remaining reads were aligned to target sequences (exon 19, 20 and 21 of *EGFR* gene) with bwa (version 0.6.2) using the bwasw mode for aligning long reads [26] and parameter setting “-b5 -q2 -r1 -z10”. 

#### Estimation of variant/error rate

Using samtools (version 0.1.18) [27], the generated mapping data by bwa (SAM files) were converted to BAM files and processed to obtain the per base coverage (pileup files). Subsequently, we summarized the base counts for each target base position (e.g., *EGFR* codons 790 and 858) using an in-house-devised perl script. Frequencies of variants/errors (substitutions) were calculated by dividing base counts of substitutions by all base counts on each position. Because of a high error rate for insertions/deletions, detection of several base-pair deletions in exon 19 was difficult. Instead, we aligned reads to eight template sequences corresponding to major deletion types in the COSMIC database of the Wellcome Trust Sanger Institute (http://www.sanger.ac.uk/genetics/CGP/cosmic/), using bwa. The nucleotide positions for these deletions in the human genome (GRCh37/hg19) are 55242463-55242477, 55242465-55242476, 55242465-55242479, 55242466-55242480, 55242466-55242486, 55242468-55242482, 55242469-55242477, and 55242469-55242486 in chr7, and the cDNA positions (NM_005228) are 2233-2247, 2235-2246, 2235-2249, 2236-2250, 2236-2256, 2238-2252, 2239-2247, and 2239-2256 (position 1 refers to the A in the ATG start codon). The frequencies of the deletion mutations were estimated by dividing the number of reads aligned to deletion-type sequences by the number of all reads aligned to exon 19 sequences with or without a deletion. Because incomplete matches with deletion-type template sequences were observed due to the diversity of deletions [28], we employed the deletion type with the maximum number of aligned reads for the estimation of mutation frequency.

### Statistical analysis

For each nucleotide substitution pattern, parameters of Poisson and negative binomial distribution were estimated using the method of moments with data from 48 normal DNA samples. Parameters *lambda* and *r* were rounded up to integer values. Calculations based on these probability distributions were executed using Microsoft Excel 2011 and Casio’s high accuracy calculation service (http://www.keisan.com). 

### Ethics statement

This study was approved by the ethic committee of Osaka Medical Center for Cancer and Cardiovascular Diseases. Written informed consent was obtained from all patients recruited in this study. 

## Supporting Information

File S1 Figure S1. Relationship between the averages and variances of the insertion/deletion errors. Figure S2. Outline of the method. Figure S3. Dynamics of mutant alleles in plasma cell-free DNA during EGFR-TKI treatment.
(PDF)

Table S1 **Relationship between the error rate, read depth, and detection limit, assuming that the read error follows a Poisson distribution.**
(XLS)

Table S2 **Raw data for Figure 1b.**
(XLS)

Table S3 **Raw data for Figure 1c.**
(XLS)

Table S4 **Base changes detected in the target loci in 155 retrospective samples.**
(XLS)

Table S5 **List of primers with index and adaptor.**
(XLS)
