# Supplementary material for: Quantitative Identification of Mutant Alleles Derived from Lung Cancer in Plasma Cell-Free DNA via Anomaly Detection Using Deep Sequencing Data
Source: PLoS One. 2013 Nov 21;8(11):e81468. doi: 10.1371/journal.pone.0081468 (PMC3836767; doi:10.1371/journal.pone.0081468)
Supplement: File S1 — Figure S1. Relationship between the averages and variances of the insertion/deletion errors. Figure S2. Outline of the method. Figure S3. Dynamics of mutant alleles in plasma cell-free DNA during EGFR-TKI treatment. (PDF) [file pone.0081468.s001.pdf]

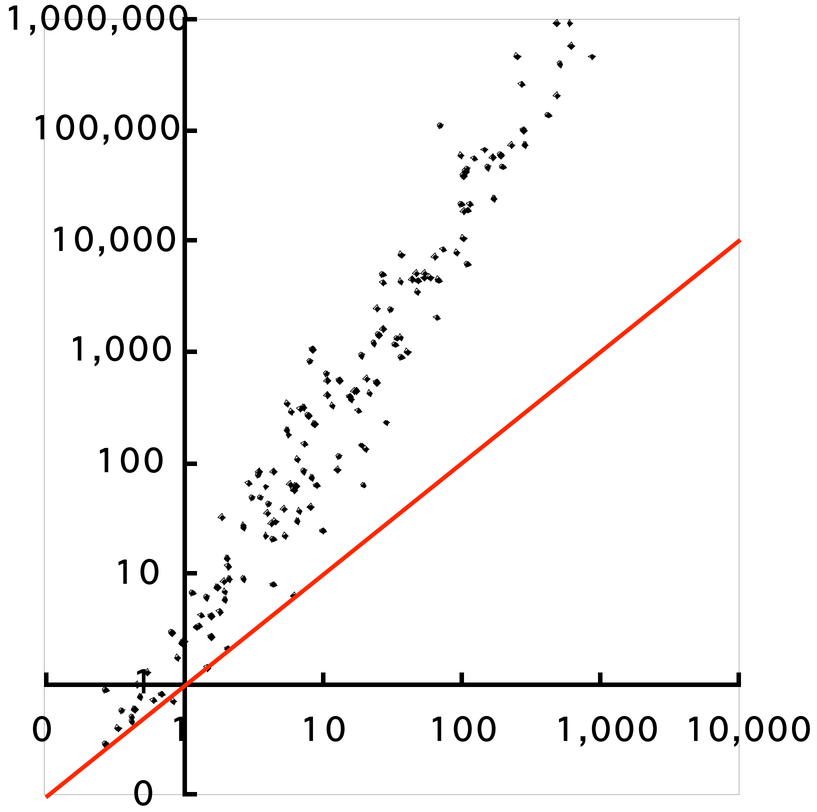

Figure S1. Relationship between the averages and variances of the insertion/deletion errors, presented as the number in 100,000 reads. Horizontal axis, average; vertical axis, variance.

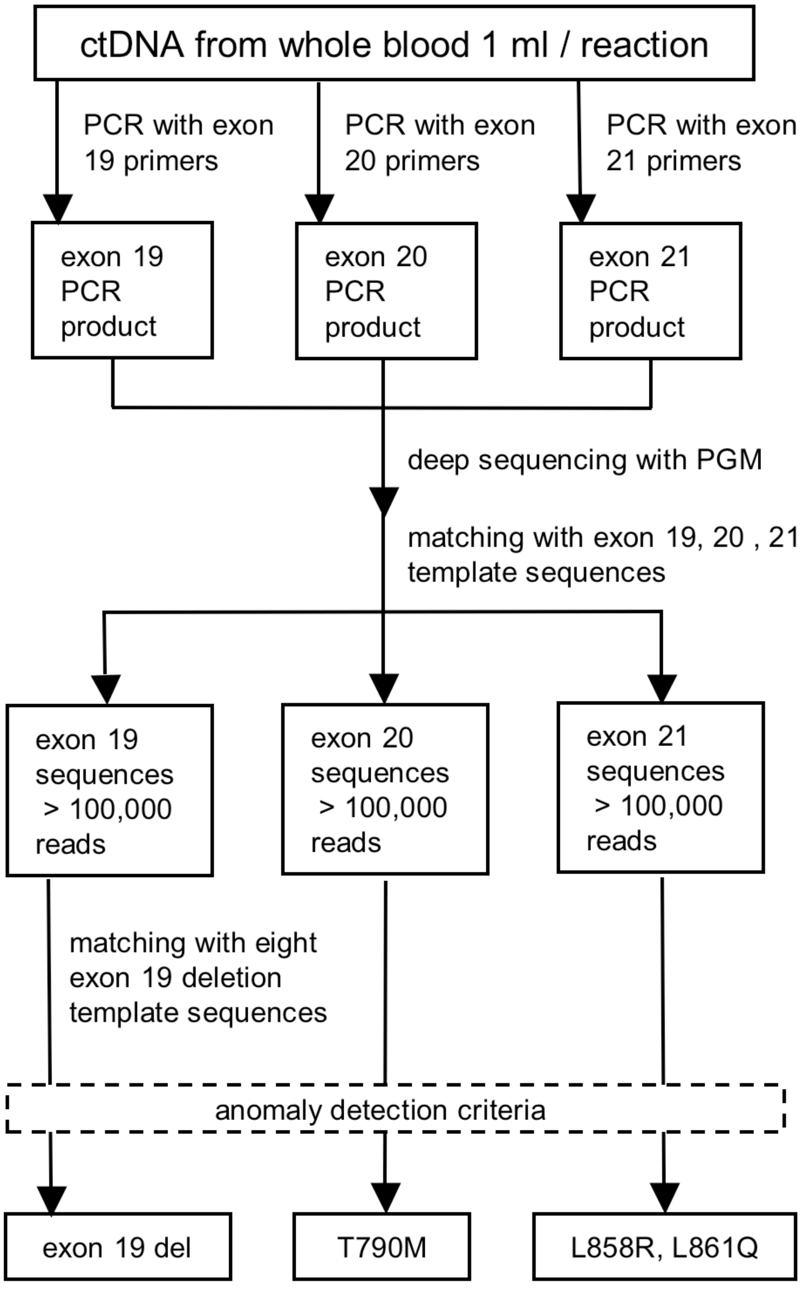

Figure S2. Outline of the method.

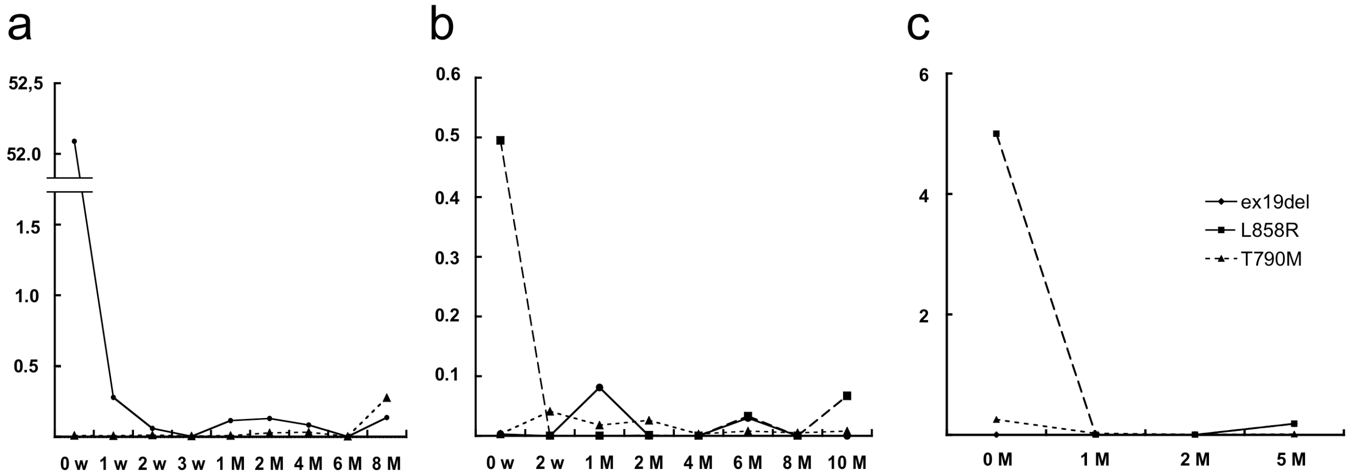

Figure S3. Dynamics of mutant alleles in plasma cell-free DNA during EGFR-TKI treatment. a, Patient 226, b, Patient 243, c, Patient 41. Horizontal axis, time point after the initiation of gefitinib treatment (a, b) or erlotinib-pemetrexed treatment (c); vertical axis, mutant allele frequency (%).
